# Functionalized ionic liquids based on vegetable oils for rare earth elements recovery†

**DOI:** 10.1039/d0ra00448k

**Published:** 2020-07-16

**Authors:** Fujian Li, Jingyao Zeng, Xiaoqi Sun

**Affiliations:** CAS Key Laboratory of Design and Assembly of Functional Nanostructures, Fujian Institute of Research on the Structure of Matter, Chinese Academy of Sciences Fuzhou 350002 PR China xqsun@fjirsm.ac.cn; CAS Key Laboratory of Green Process and Engineering, State Key Laboratory of Multiphase Complex Systems, Beijing Key Laboratory of Ionic Liquids Clean Process, Institute of Process Engineering, Chinese Academy of Sciences Beijing 100190 PR China; Ganzhou Rare Earth Group Co., Ltd., China Southern Rare Earth Ganzhou 341000 PR China; Jiangxi University of Science and Technology Ganzhou 341000 PR China

## Abstract

Functionalized ionic liquids (FILs) based on vegetable oils have been directly synthesized and used for the first time to extract rare earth elements (REEs). Carbon dioxide gas is introduced to successfully strip REE-loaded FILs in the presence of water. The novel extraction process reveals some advantages of accessibility, biocompatibility and sustainability as well as cost efficiency.

## Abstract 2

Vegetable oil based ionic liquids for rare earth recovery.

## Graphical abstract

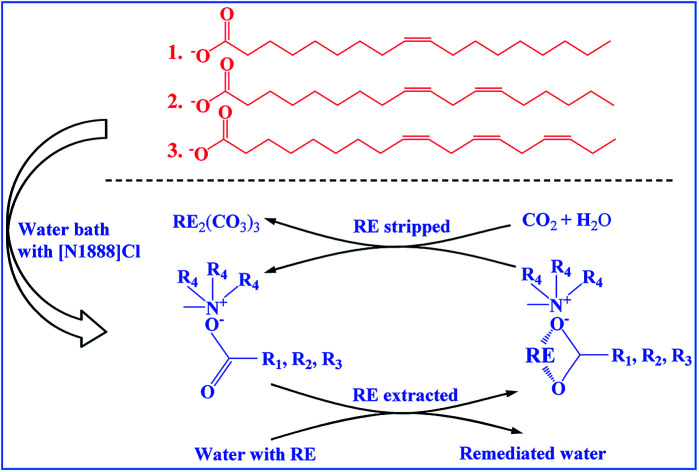

REEs (La–Lu, Y and Sc) and their compounds are critical materials in computers, audio systems, electric and hybrid vehicles, cell phones, wind turbines, magnetic resonance imaging machines, *etc.*^1–3^ However, many of these elements are indeed rare and geologically often unevenly distributed.^3,4^ The extraction of REEs from aqueous solution is an extremely important topic in the world today. It is anticipated to be of even greater importance due to the increasing demand for these critical metals. Unfortunately, there is a problem of economical extraction in producing REEs. Also, the extraction process requires large volumes of harmful chemicals. Accordingly, some improvements are sorely needed.^1^

Extractant is important to reduce the cost as well as ecological impact in the extraction and separation process of REEs. Recently, a series of novel functionalized ionic liquids (FILs) have been designed,^5,6^ which could be used directly as the extracting phase for REEs. The FILs contributed to overcoming the problem of extractant/diluent miscibility and facilitating solvent recovery.^7–11^ Generally, these FILs were provided with a functional group of carboxylic acid,^12–15^ phosphinic acid,^16,17^ thiourea,^18^ thioether,^19^ urea^20^ or thiol,^21^ which favoured their interactions with metal ions. Apart from avoiding volatile organic compounds (VOCs), the FILs were also designed to address the issue of adding sodium hydroxide for saponification and hydrochloric acid for stripping,^15^ which revealed positive environmental impacts for REEs separation.^22^ In compliance with the basic principles of green chemistry, the optimization of the extraction process should be investigated in parallel with the minimization of (eco)toxicological hazard of chemicals. FILs are non-volatile and less toxic, but still mostly based on harmful chemicals, such as fluorinated organic materials.^23^ Additionally, another disadvantage of common FILs was their extended synthesis and purification, which made the production of FILs expensive.^24^ Industrial applications of FILs for REEs extraction should be urgently accelerated by lowering the cost and environmental impact of FILs preparation.

Recently, oleate-based FILs have been reported to extract metals by several researchers,^12,13^ which revealed excellent extraction efficiencies at the given experimental conditions. The cheaper precursors, lower water solubilities, and lower viscosities make these FILs suitable for metal extraction. Oleic, linoleic and linolenic acids, the most common carboxylic acids, are widely owned by vegetable oils with the contents up to 80 mol%.^25,26^ Nevertheless, the extraction and purification of these fatty acids from natural oils is a challenging task, which required pre-treatment steps, saponification, liquid–liquid extraction and chromatography, resulting cost inefficiently and generating volumes of chemical waste inevitably.^27^ Interestingly, heavy metal ions in aqueous solution could be successfully removed by thioether-functionalized vegetable oils, based on the rich unsaturated fatty acids.^28^ But using toxic sulphur compounds as precursors of the resultant ligands run counter to the principles of green chemistry. Also, regeneration of the absorbent needs complicated steps. The direct use of vegetable oils as precursors to synthesize FILs may be a considerable approach to a greener metal extraction process.

In this work, one-pot syntheses of VOFILs (vegetable oils based FILs) used for REEs extraction and separation, is first reported. Four common vegetable oils, *i.e.*, peanut oil ([PO]), rapeseed oil ([RO]), sunflower seed oil ([SO]) and flaxseed oil ([FO]), are introduced as examples to provide fatty acids (Table 1). Chromatograms of the four vegetable oils can be seen in Fig. S1–S4.† The FILs were synthesized by mixing saponified vegetable oils and methyltrioctylammonium chloride in one-pot water bath, named as [methyltrioctylammonium][peanut oil] ([N1888][PO]), [methyltrioctylammonium][rapeseed oil] ([N1888][RO]), [methyltrioctylammonium][sunflower seed oil] ([N1888][SO]) and [methyltrioctylammonium][flaxseed oil] ([N1888][FO]). The anion RCOO^−^ group exhibits stronger binding strength to REEs, while the cation methyltrioctylammonium provides excellent hydrophobicity (structure can be seen in Fig. S5–S7†). The synthesized FILs and REEs aqueous solution were equilibrated and stirred for 20 min in a vibrating mixer at 25 °C and then centrifuged for 10 min at 4000 rpm. The viscosities of FILs in different temperatures were given in Fig. S8,† which indicates that the viscosities of [N1888][PO], [N1888][SO] and [N1888][FO] (about 300 mPa s) are much lower than [N1888][NA] (688 mPa s) and [N1888][Cl] (1430 mPa s) at 30 °C. As shown in Fig. 1, REEs are extracted by the proposed FILs. Then, REEs loaded on the FILs are stripped and recycled using carbon dioxide and water (apparatus can be seen in Fig. S5†). Simultaneously, FILs are regenerated and used in the next cycle (Fig. 1). To evaluate the extraction efficiency of proposed FILs, methyltrioctylammonium naphthenic acid ([N1888][NA]) is synthesized with the same procedure and used as control group, considering NA extraction is the common industrial process to obtain yttrium products^29^ (more details can be seen in ESI†).

**Table 1:** Main fatty acid composition of vegetable oils (mol%)

Fatty acid	[PO]	[RO]	[SO]	[FO]
Palmitic (C16 : 0)	9.75	4.01	5.67	5.17
Stearic (C18 : 0)	5.28	2.47	5.87	5.13
Oleic (C18 : 1)	47.87	47.62	21.07	17.43
Linoleic (C18 : 2)	28.87	17.56	64.29	16.82
Linolenic (C18 : 3)	0.79	14.11	0.36	53.79
The rest	7.44	14.23	2.74	1.66

**Fig. 1:**
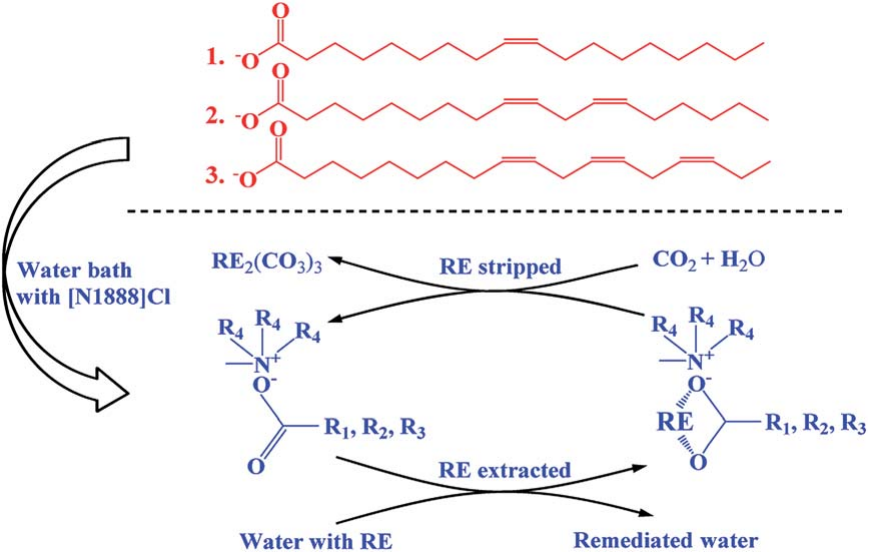
FILs based on vegetable oils for REEs recovery.

As the extraction mechanism of FILs for REE(III) was proposed as ion association,^15^ the extraction process of VOFILs in this study can be written as the following eqn (1):

1 2[RECl_3_](aq) + 3[VOFIL](org) = [RECl_3_]_2_[VOFIL]_3_(org)

The extraction efficiency (*E*) was determined by mass balance in eqn S(1), ESI.† Extraction performances are shown in Fig. 2. Because the effective extractant in organic phase is reduced from 100% to 20% by volume, the loaded REEs on FILs is significantly decreased as lowering the FILs concentration. The tendency is similar to both of VOFILs and [N1888][NA]. The four VOFILs with no diluent extract about 0.152 mol L^−1^ REEs, a little smaller than that of [N1888][NA] (0.187 mol L^−1^). The main reason lies in naphthenic acid (structure can be seen Fig. S9†) used herein has a purity of 99%, comparing that the long-chain carboxylic acid content is about 85%. Interestingly, the maximal loading amounts of VOFILs for REEs are about 81–85% of that by using [N1888][NA], fitting with the content of effective extractants. In other word, it can be improved by purifying the used carboxylic acids from vegetable oils to get higher loading amounts.

**Fig. 2:**
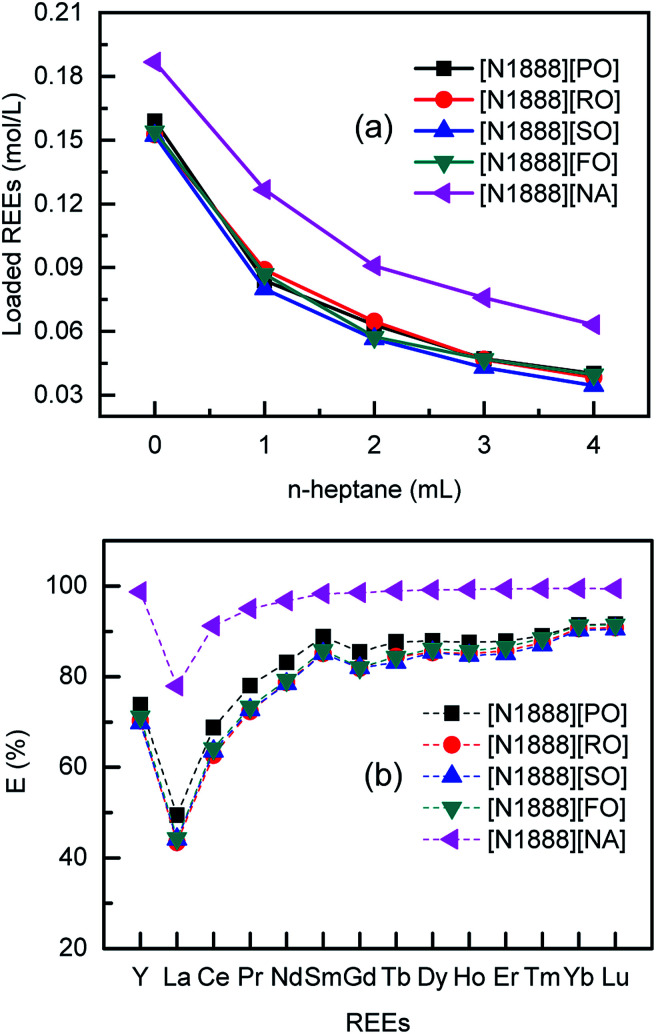
Extraction efficiency of mixed REEs by FILs. (a) FILs concentration affection, organic phase: 1 mL FILs diluted in *n*-heptane; (b) extraction sequence, organic phase: FILs with no diluent. Aqueous phase: ∑REEs = 0.098 M (0.007 M each RE ion), pH = 4.176; A/O = 2 : 1.

It can be observed that the pH values of aqueous solutions are increased during the REEs extraction processes. After adding acid–base indicator of methyl red and bromocresol green, the feed REEs solution looks dull-red. While all the five raffinates appear pale green which are similar to the colour of pure water sample (Fig. S10†). It indicates that VOFILs also extracted H^+^ during REEs extraction process, causing pH increased.^13^ As for the extraction sequence in Fig. 2, the extraction efficiency of REEs increase continuously from lanthanum to lutetium (atomic number from 57 to 71). The behaviour of yttrium most closely resembles that of the lighter lanthanides (praseodymium or neodymium). The extraction order of VOTILs for REEs follows the approximate reverse sequence, *i.e.*, Lu, Yb, Tm, Er, Ho, Dy, Tb, Gd, Sm, Nd, Pr, Y, Ce, La. It reveals that VOFTLs may be also used for purification and separation of individual rare earth from the lanthanide mixture due to the considerable selectivity, similar to conventional extractants such as P507 (2-ethyl(hexyl) phosphonic acid mono-2-ethylhexyl ester), P204 (di(2-ethylhexyl)phosphate) and NA (naphthenic acid), *etc.* It should be mentioned that DESs (deep eutectic solvents),^24,30,31^ prepared by plant extracts such as decanoic acid and lauric acid, also revealed the advantages of accessibility, biocompatibility and sustainability for metal extraction. But the extractability of VOFILs is 10 times larger than that of DESs, which means the former is more suitable for metal enrichment while the latter is usually designed to remove trace metal ions. Moreover, the studied VOFILs in this communication may be cheaper than DESs.^24,30,31^ Because VOFILs were directly prepared from vegetable oils, while the DES were synthesized by plant extracts.

Regeneration and reuse of FILs are highly desired for their industrial applications. Aqueous HCl solution is common used for REEs stripping from extractant. However, large amount of acid used as stripping agent is not desired from an economical and environmental point of view. The precipitation stripping using oxalic acid^13,32^ and carbonic acid^33^ was developed. Herein, carbon dioxide and water, which can formed as carbonic acid under given conditions (apparatus can be seen in Fig. S11†), are introduced to strip REEs.

As the extraction mechanism of FILs for REE(III) was proposed as ion association,^15^ the stripping process in the case of carbon dioxide and water can be written as following eqn (2):2
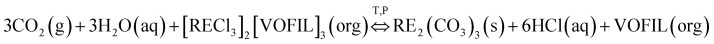


In this case, the REEs formed stronger water insoluble complexes (RE_2_(CO_3_)_3_) that precipitated out of the FILs. The organic samples (before and after CO_2_ stripping) were equilibrated with 6 mol m^−3^ hydrochloric acid solution to strip the loaded REEs completely and the aqueous solutions were analysed.^34^ The stripping efficiency was close relevant to the concentration of H_2_CO_3_ in aqueous phase. Herein, dissociation equilibrium of H_2_CO_3_ was sensitive to the partial pressure of carbon dioxide (*P*_CO_2__) and the operating temperature based on the solubility of carbon dioxide in water.^35^ Therefore, processing parameters of *P*_CO_2__ and temperature were chosen to evaluate the stripping efficiency in this paper.

REEs loaded [N1888][SO] was chosen as an example for testing stripping efficiency. The CO_2_ stripping efficiency (*S*%) is given as eqn S(2) in ESI.† As shown in Fig. 3, the *S*% is achieved to 99.5% under the carbon dioxide pressure of 3 MPa at 30 °C. However, it decreases to 50%–66.5% in the condition of 0.5 MPa and 60 °C. It can be concluded that increasing *P*_CO_2__ and decreasing operating temperature heighten the stripping efficiency, which goes well with the direction of increasing H_2_CO_3_ concentration in stripping solution. Considering the cost and security of reaction equipment, *P*_CO_2__ of 3 MPa at 30 °C is suitable for efficient stripping.

**Fig. 3:**
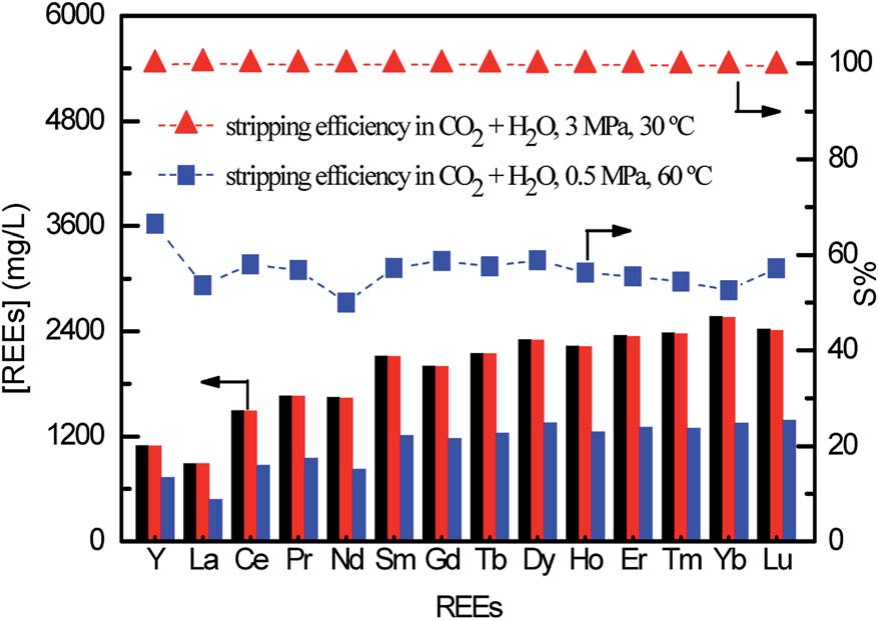
REEs stripping efficiency by H_2_CO_3_, black column: initial REEs loaded on FILs, red column: REEs stripped under 3 MPa and 30 °C, blue column: REEs stripped in 0.5 MPa and 60 °C.

Fig. 4 shows the phase interfaces of O/A and A/S after centrifugation, indicating that the regeneration of VOFILs and recycling of RE_2_(CO_3_)_3_ can be achieved in this combined stripping and precipitation step. It is worthwhile to mention that there are still REEs in aqueous phase (857.5 mg L^−1^, Table S1†) due to the chemical decomposition of RE_2_(CO_3_)_3_ by H^+^ from H_2_CO_3_ and HCl. The residual REEs could be simply precipitated by adjusting pH of the solution. Finally, REO products were obtained by roasting RE_2_(CO_3_)_3_.

**Fig. 4:**
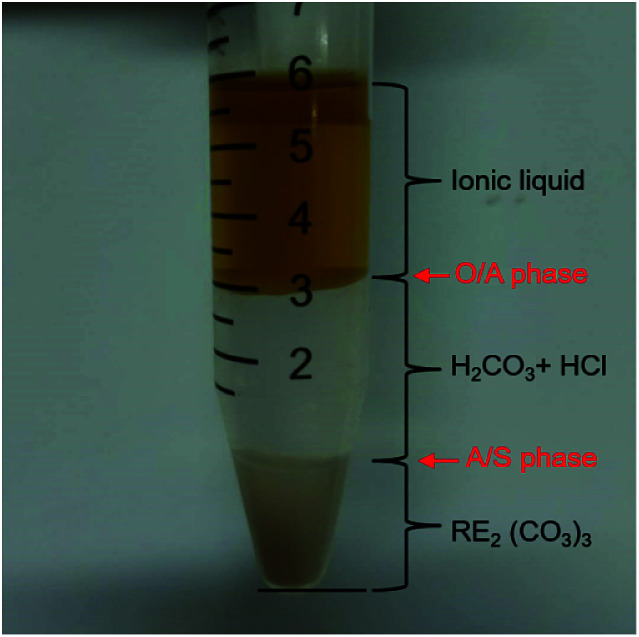
REEs Loaded FILs stripped by CO_2_ + H_2_O.

In summary, this communication represents the first protocol of REEs extraction using vegetable oils based FILs, providing the possibilities of accessibility, biocompatibility and sustainability for metal extraction. The extraction capacity is as high as 0.15 mol L^−1^, which is decreased with the concentration of effective extractant is decreased. The extraction order follows the approximate reverse sequence, *i.e.*, Lu, Yb, Tm, Er, Ho, Dy, Tb, Gd, Sm, Nd, Pr, Y, Ce, La. Carbon dioxide and water are introduced to successfully strip REEs loaded VOFILs at 3 MPa, 30 °C. The stripping efficiencies of REEs are markedly increased with increasing carbon dioxide pressure and decreasing operating temperature. Only using vegetable oil based VOFILs, CO_2_ and H_2_O, the extraction and stripping strategy reveals sustainable potential in REEs recovery.

## Conflicts of interest

There are no conflicts to declare.

## Supplementary Material

RA-010-D0RA00448K-s001
