# Supplementary material for: Functionalized ionic liquids based on vegetable oils for rare earth elements recovery
Source: RSC Adv. 2020 Jul 16;10(45):26671–4. doi: 10.1039/d0ra00448k (PMC9055440; doi:10.1039/d0ra00448k)
Supplement: RA-010-D0RA00448K-s001 [file RA-010-D0RA00448K-s001.pdf]

Electronic Supplementary Material (ESI) for RSC Advances.
This journal is © The Royal Society of Chemistry 2020

Electronic Supplementary Information (ESI) for RSC Advance.

This journal is © The Royal Society of Chemistry 2020

SUPPORTING INFORMATION

Functionalized ionic liquids based on vegetable oils for rare earth elements recovery

Fujian Li[a,b], Jingyao Zeng[d] and Xiaoqi Sun[a,c]*

a. CAS Key Laboratory of Design and Assembly of Functional Nanostructures, Fujian Institute of Research on the Structure of Matter, Chinese Academy of Sciences, Fuzhou, 350002, PR China.

b. CAS Key Laboratory of Green Process and Engineering, State Key Laboratory of Multiphase Complex Systems, Beijing Key Laboratory of Ionic Liquids Clean Process, Institute of Process Engineering, Chinese Academy of Sciences, Beijing, 100190, PR China.Ganzhou Rare Earth Group Co., Ltd., China Southern Rare Earth, Ganzhou, 341000, PR China

c. Ganzhou Rare Earth Group Co., Ltd., China Southern Rare Earth, Ganzhou, 341000, PR China

d. Jiangxi University of Science and Technology, Ganzhou, 341000, PR China

*Corresponding author: xqsun@fjirsm.ac.cn.

Methodology

Materials and reagents

Methyltrioctylammonium bromide had a purity of 99%. Sodium hydroxide, and ethanol (70%) was purchased from Sigma-Aldrich. YCl_3 (99.999%), $LaCl_3$ (99.999%), $CeCl_3$ (99.99%), $PrCl_3$ (99.9%), $NdCl_3$ (99.999%), $SmCl_3$ (99.9%), $GdCl_3$ (99.99%), $TbCl_3$ (99.99%), $DyCl_3$ (99.99%), $HoCl_3$ (99.9%), $ErCl_3$ (99.9%), $TmCl_3$ (99.9%), $YbCl_3$ (99.99%) and $LuCl_3$ (99.999%) solutions were kindly provided by China Southern Rare Earth Group Co., Ltd., (China). Also, naphthenic acid (NA), n-heptane, hydrochloric acid (HCl) with chemical grade were all obtained from China Southern Rare Earth Group Co., Ltd., (China). CO_2 was purchased from Shandong construction high pressure vessel Co. LTD. Peanut oil (PO), rapeseed oil (RO), sunflower seed oil (SO) and flaxseed oil (FO) were purchased from Guoguang Super Market, Ganzhou city (China). All the chemicals were used as received without any additional purification step.

Analysis

The concentrations of REEs in aqueous phase were determined by Inductively Coupled Plasma Optical Emission Spectroscopy (ICP-OES) Horiba Ultima 2. The composition of vegetables oil was determined with GC-MS and the spectra were recorded on an Agilent 7890-GC, Jeol AccuTOF GCV-MS (GC-MS conditions for fatty acid analysis followed as GB5009.168-2016 described[1]).

In 25-mL round-bottom flasks, 10 mg of samples were added to 3 mL sodium methylate solution with phenolphthalein. The reaction medium was refluxed for 10 min; 3 mL of chlorhydric methanol was added to phenolphthalein discoloration. The mixture was refluxed again for 10 min and then cooled to ambient temperature; 8 mL hexane and 10 mL water were added, and the organic phase was recovered, dried over anhydrous sodium sulfate and filtered for subsequent GC analysis: Agilent 6890 series using a CP-Sil88, 100m×0.25mm×0.2μm; 1.0μl Fatty acid methyl esters were directly injected into the GC. Carrier gas: helium flow rate 1.8 mL/min, splitting ratio: 20, injector temperature: 230°C, FID detector temperature: 250°C. The temperature settings were as follows: 45°C for 4min, 45-175°C at 13°C/min, 175°C for 27min, 175-215°C at 4°C/min, 215°Cfor 35 min.

FILs synthesis

FILs were synthesized in one-pot water bath followed as the method of the paper[2].

1. Synthesis of methyltrioctylammonium peanut oil ([N1888][PO])

Sodium hydroxide (0.497g) was added to peanut oil (5.56ml) dissolved in ethanol 70% (50mL). The reaction mixture was stirred overnight at room temperature. Sodium oleate (4.52g) was obtained as a white soap after removal of the solvent under vacuum. Afterwards, methyltrioctylammonium (7.194g) dissolved in water (50mL) was added to the attained soap. The reaction mixture was stirred for 3h at room temperature. The water phase was separated and the organic phase was washed with water (3 x 50mL). A viscous product was obtained after removal the solvent (11.566g).

2. Synthesis of methyltrioctylammonium rapeseed oil ([N1888][RO])

Sodium hydroxide (0.497g) was added to rapeseed oil (5.56ml) dissolved in ethanol 70% (50mL). The reaction mixture was stirred overnight at room temperature. Sodium oleate (4.76g) was obtained as a white soap after removal of the solvent under vacuum. Afterwards, methyltrioctylammonium (7.194g) dissolved in water (50mL) was added to the attained soap. The reaction mixture was stirred for 3h at room temperature. The water phase was separated and the organic phase was washed with water (3 x 50mL). A viscous product was obtained after removal the solvent (11.8591g).

3. Synthesis of methyltrioctylammonium sunflower seeds oil ([N1888][SO])

Sodium hydroxide (0.497g) was added to seeds oil (5.56ml) dissolved in ethanol 70% (50mL). The reaction mixture was stirred overnight at room temperature. Sodium oleate (5.22g) was obtained as a white soap after removal of the solvent under vacuum. Afterwards, methyltrioctylammonium (7.194g) dissolved in water (50mL) was added to the attained soap. The reaction mixture was stirred for 3h at room temperature. The water phase was separated and the organic phase was washed with water (3 x 50mL). A viscous product was obtained after removal the solvent (12.0805g).

4. Synthesis of methyltrioctylammonium flaxseed oil ([N1888][FO])

Sodium hydroxide (0.497g) was added to flaxseed oil (5.56ml) dissolved in ethanol 70% (50mL). The reaction mixture was stirred overnight at room temperature. Sodium oleate (5.02g) was obtained as a white soap after removal of the solvent under vacuum. Afterwards, methyltrioctylammonium (7.194g) dissolved in water (50mL) was added to the attained soap. The reaction mixture was stirred for 3h at room temperature. The water phase was separated and the organic phase was washed with water (3 x 50mL). A viscous product was obtained after removal the solvent (12.2604g).

5. Synthesis of methyltrioctylammonium naphthenic acid ([N1888][NA])

Sodium hydroxide (0.497g) was added to naphthenic acid (3.892ml) dissolved in ethanol 70% (50mL). The reaction mixture was stirred overnight at room temperature. Saponified naphthenic acid (2.85g) was obtained as a white soap after removal of the solvent under vacuum. Afterwards, methyltrioctylammonium (7.194g) dissolved in water (50mL) was added to the attained soap. The reaction mixture was stirred for 3h at room temperature. The water phase was separated and the organic phase was washed with water (3 x 50mL). A viscous product was obtained after removal the solvent (9.544g).

Metal extraction and stripping procedure

Firstly, the REEs solutions were prepared by diluting or mixing individual $RECl_3$ solution. The required pH value of aqueous phase was adjusted with HCl or NaOH solution. The ILs synthesized were tested for the mixed REEs solution extraction. One ml of the selected ILs was added to two ml of metal solution in the centrifuge tubes. The samples were stirred for 20 min in a vibrating mixer and afterwards centrifuged for 10 min at 4000 rpm. Afterwards, aqueous phases were collected and analyzed by inductively coupled plasma (ICP).

The extraction efficiency (E) was determined by mass balance as following:

$$E\% = \frac{[M]_{aq,ini} - [M]_{aq,eq}}{[M]_{aq,\ ini}} \times 100\% \qquad Eq(1)$$

where $[M]_{aq,\ ini}$ and $[M]_{aq,\ eq}$ (determined by ICP) represent the initial and equilibrium metal concentration in the aqueous phase, respectively. Metals loaded on VOFILs can be determined by mass balance of the reduced amount of the aqueous phase.

Acid-base indicator was prepared by 30 ml bromocresol-green Ethyl Alcohol solution (0.1 Vol%) and 10 ml Methyl-red Ethyl Alcohol solution (0.1 Vol%) due to the narrow colour change interval at pH = 5.1. The REEs feed solution and reffinates were diluted by one ml of ten ml pure water and added the prepared indicator.

The stripping efficiency was determined by mixed with the metal loaded FILs and CO_2 in the presence of water in the apparatus, with the chosen pressure of carbon dioxide (P_{CO2}) and the operating temperature (Figure S6.). Also, the organic phases (before and after CO_2 stripping) were mixed with 6 mol/m^3 hydrochloric acid solution to strip completely REEs in the organic phase, and the aqueous solutions were analyzed. CO_2 stripping efficiencies were determined by the following equation.

$$S\% = \frac{[M]_{bef} \times V_{HCl,\ bef} - [M]_{aft} \times V_{HCl,aft}}{[M]_{bef} \times V_{HCl,bef}} \times 100\% \qquad Eq(2)$$

where $[M]_{bef}$ and $[M]_{aft}$ (determined by ICP) represent the REEs concentration stripped by 6M HCl stripping aqueous before and after CO_2 stripping, respectively. $V_{HCl,\ bef}$ and $[M]_{HCl,\ aft}$ represent the volume of used 6M HCl stripping aqueous before and after CO_2 stripping, respectively.

Figures

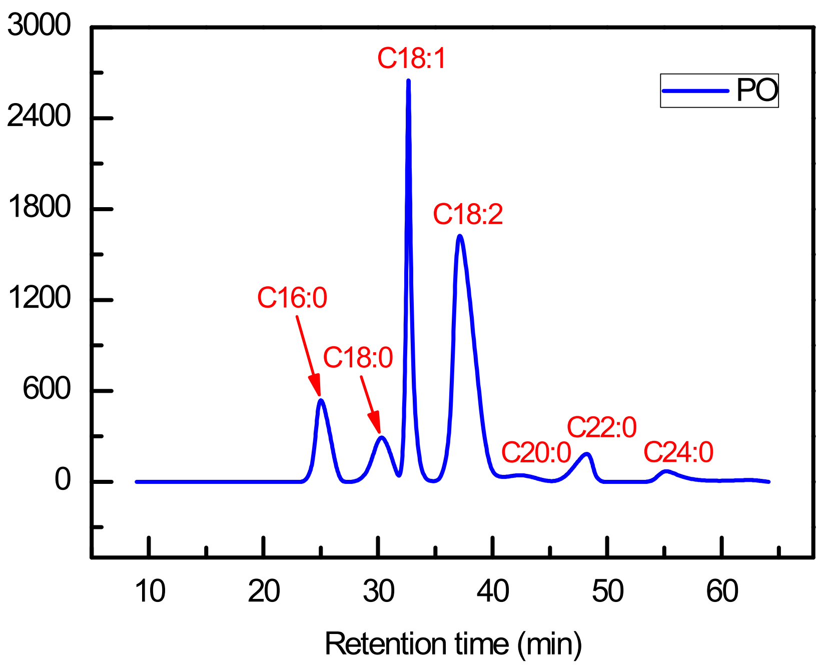

Figure S1. Chromatograms of [PO]

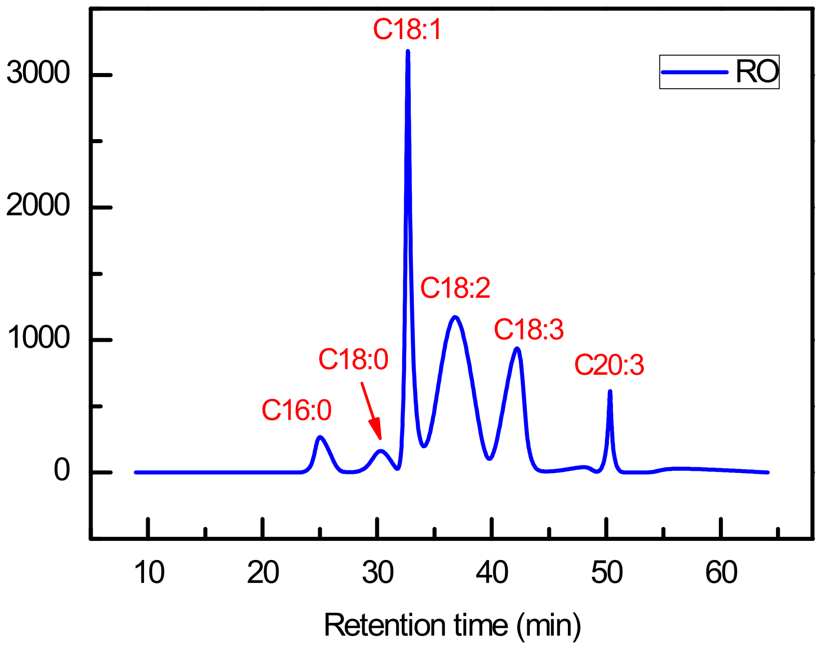

Figure S2. Chromatograms of [RO]

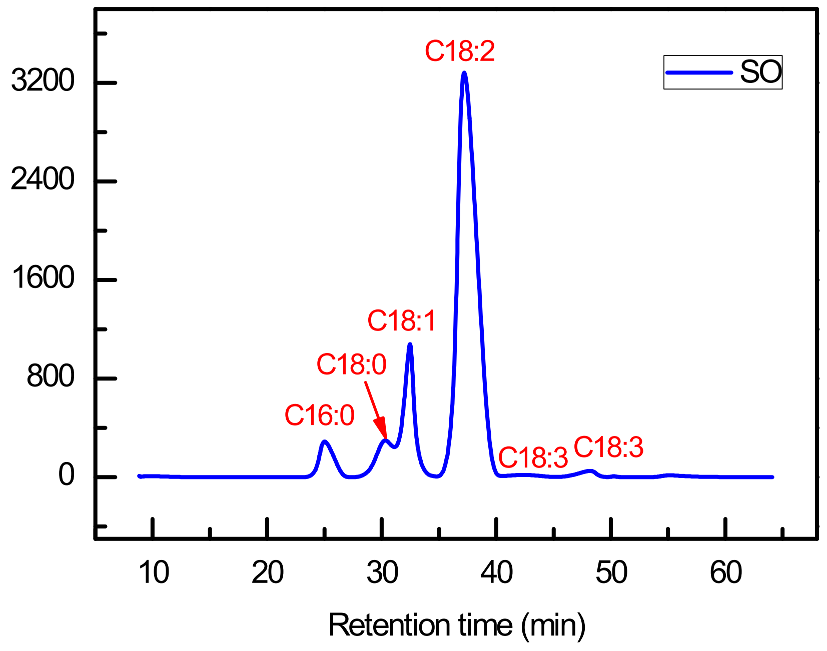

Figure S3. Chromatograms of [SO]

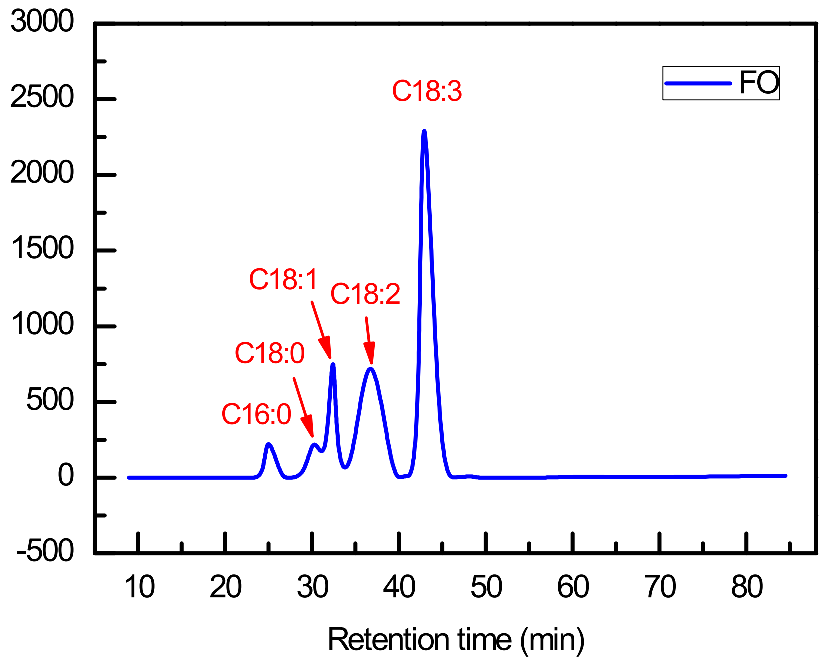

Figure S4. Chromatograms of [FO]

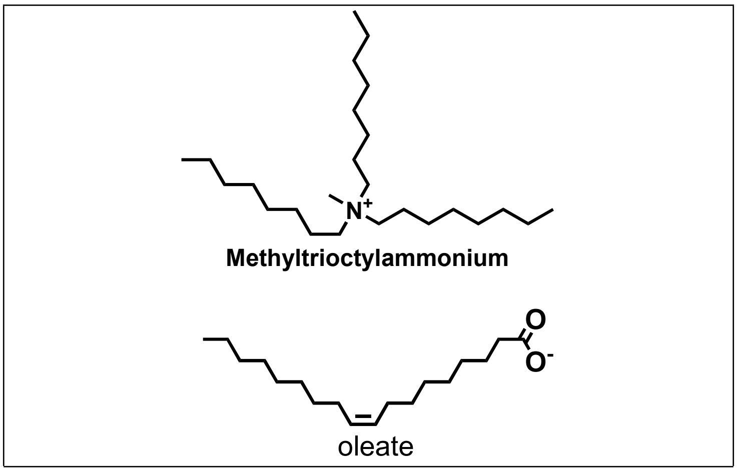

Figure S5. Structure of methyltrioctylammonium oleate

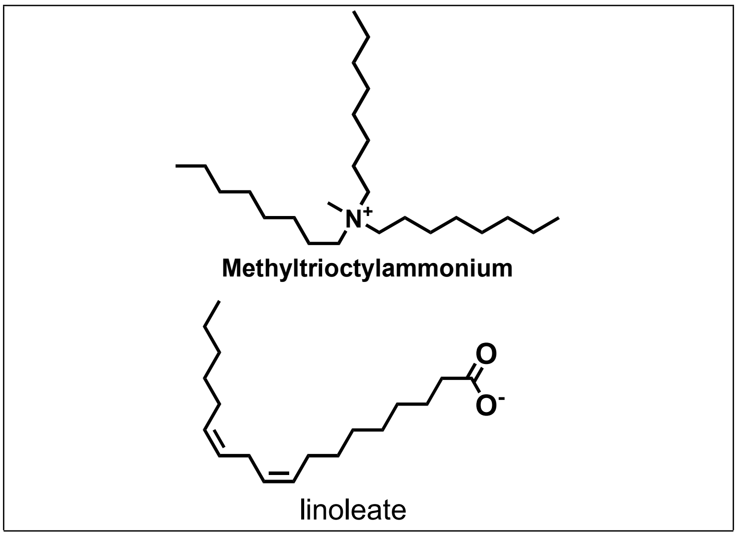

Figure S6. Structure of methyltrioctylammonium linoleate

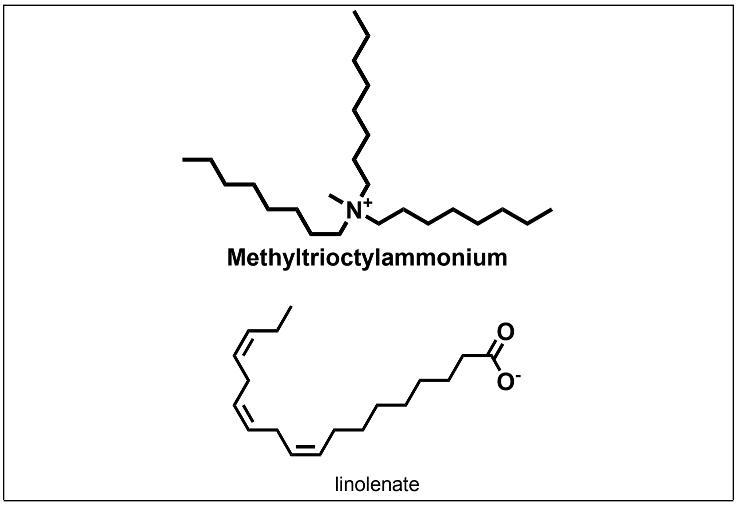

Figure S7. Structure of methyltrioctylammonium linolenate

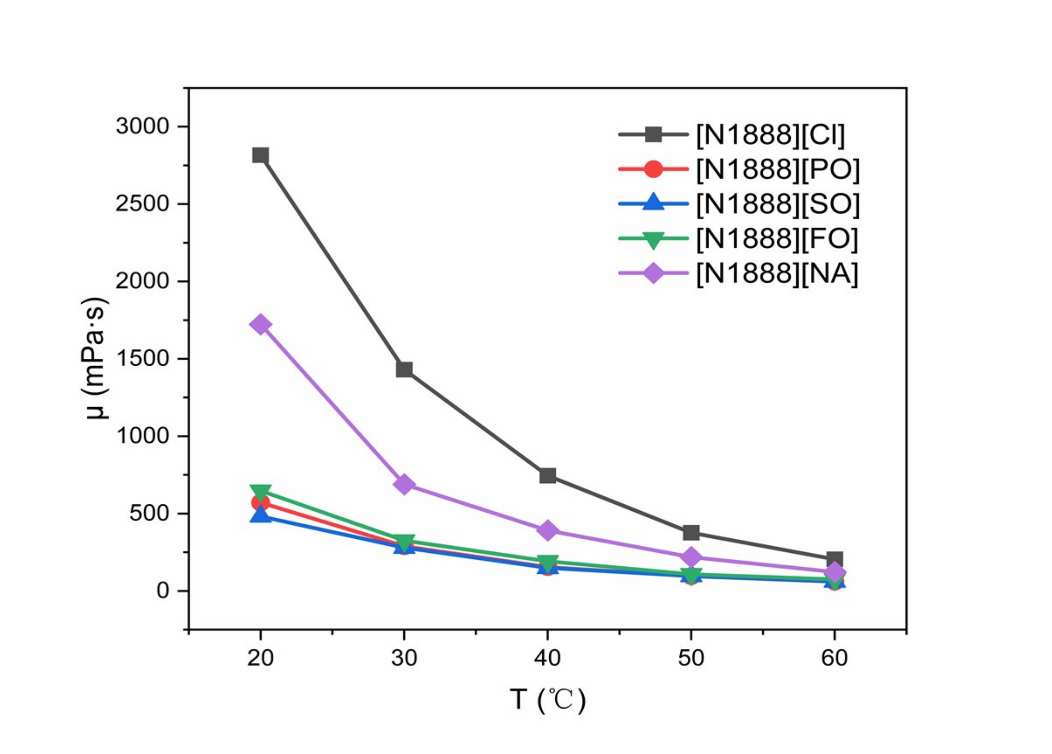

Figure S8. The viscosities of ILs in different temperature.

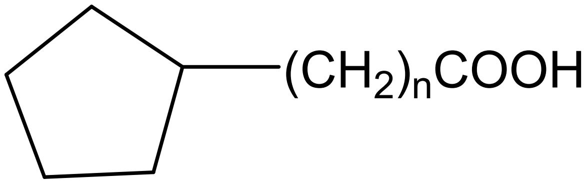

Figure S9. The structure of Naphthenic acid (NA)

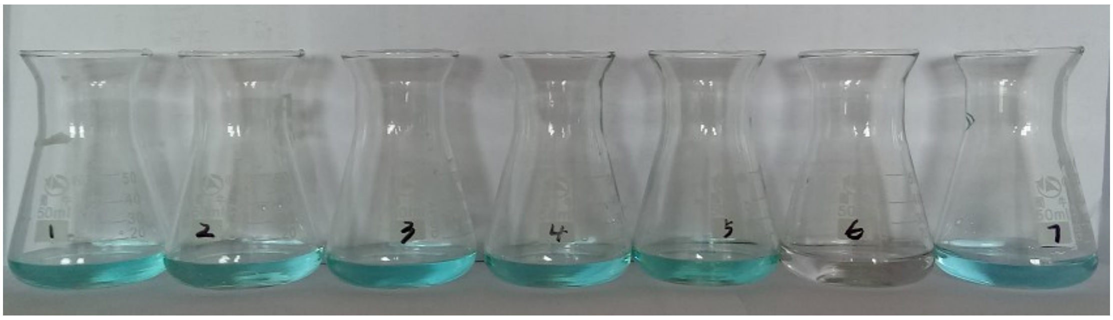

Figure S10. The change of pH in feed and raffinate. 1-5: raffinates from ([N1888][PO]), ([N1888][RO]), ([N1888][SO]), ([N1888][FO]) and ([N1888][NA]), respectively; 6: the feed solution; 7: pure water. All the samples were prepared by diluting one ml target solution of ten ml pure water.

Acid extraction equilibrium may be lies as follows:

$$[HCl](ag)+[VOFIL](org)=[HCl][VOFIL](org) \quad \text{eq(3)}$$

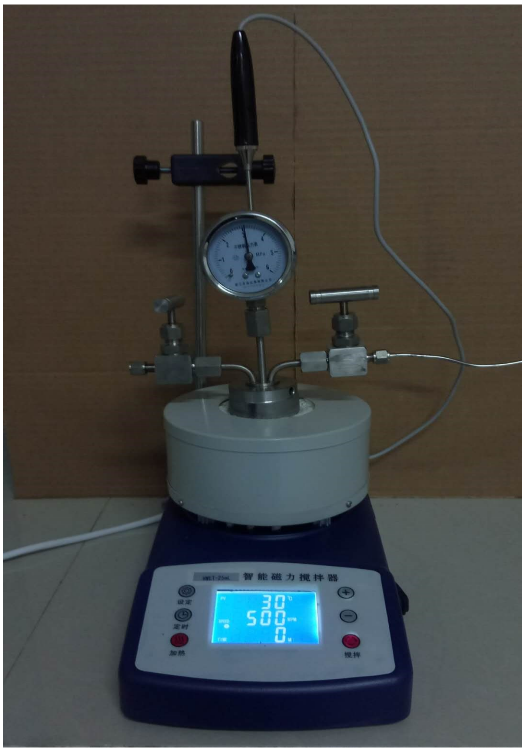

Figure S11. Apparatus of rare-earth loaded VOFILs stripped by CO_2 + H_2O

Tables

Table S1. REEs in aqueous phase after CO_2 stripping

<table>
<tr><td>Element</td><td>Y</td><td>La</td><td>Ce</td><td>Pr</td><td>Nd</td><td>Sm</td><td>Gd</td><td>Total</td></tr>
<tr><td>Content(mg/L)</td><td>97</td><td>17</td><td>18.5</td><td>19</td><td>18.5</td><td>21</td><td>43</td><td rowspan="3">857.5</td></tr>
<tr><td>Element</td><td>Tb</td><td>Dy</td><td>Ho</td><td>Er</td><td>Tm</td><td>Yb</td><td>Lu</td></tr>
<tr><td>Content(mg/L)</td><td>48</td><td>57.5</td><td>76</td><td>92.5</td><td>108.5</td><td>112.5</td><td>128.5</td></tr>
</table>

Note: REEs stripped in 3MPa and 30℃

References

1. Determination of fatty acids in food. In Chinese: GB5009.168-2016, 2017.

2. D. Parmentier, S. J. Metz and M. C. Kroon, Green Chem., 2013, 15, 205.

Glossary of abbreviations

REEs	Rare earth elements
ILs	Ionic liquids
FILs	Functionalized ionic liquids
VOFILs	Vegetable oils based functionalized ionic liquids
[N1888][NA]	[Methyltrioctylammonium][Naphthenic acid]
[N1888][PO]	[Methyltrioctylammonium][Peanut oil]
[N1888][RO]	[Methyltrioctylammonium][Rapeseed oil]
[N1888][SO]	[Methyltrioctylammonium][Sunflower seed oil]
[N1888][FO]	[Methyltrioctylammonium][Flaxseed oil]
